# Investigation of the protective effect of beta caryophyllene against indomethacin-induced gastric ulcer in rats: in vivo and in vitro study

**DOI:** 10.1007/s00210-025-04269-7

**Published:** 2025-05-22

**Authors:** Merve Bolat, Fikret Çelebi, Emin Şengül, İrfan Çınar, Serkan Yıldırım, İsmail Bolat

**Affiliations:** 1 Department of Physiology, Faculty of Veterinary Medicine, Atatürk University, Erzurum, Turkey; 2 Department of Pharmacology and Toxicology, Faculty of Medicine, Kastamonu University, Kastamonu, Turkey; 3 Department of Pathology, Faculty of Veterinary Medicine, Kyrgyzs-Turkish Manas University, Bishkek, Kyrgyzstan; 4 Department of Pathology, Faculty of Veterinary Medicine, Atatürk University, Erzurum, Turkey

**Keywords:** Apoptosis, Beta caryophyllene, ER stress, Gastric ulcer, Inflammation, Oxidative stress

## Abstract

In this study, the protective and healing effects of beta-caryophyllene (BCP) on gastric mucosa in indomethacin (INDO)-induced gastric ulcer model were investigated. In the study, 116 male Sprague–Dawley rats were used. In in vivo experiments, rat was administered doses of 50–100–200 mg/kg BCP and 5 mg/kg omeprazole for 14 days, and indomethacin (100 mg/kg) was given on the last day. In in vitro experiments, the effects of BCP (250–500–1000 µg/ml) on gastric motility and acid secretion were examined by isolated organ bath method. It was found that INDO treatment increased MDA level in gastric tissue, but decreased GPx and SOD activities. Nrf2 and HO-1 levels were decreased in INDO-treated groups. INDO increased TNF-α, IL-1β, and NF-κB levels and iNOS activity, but decreased COX-1 activity and PGE2 levels. INDO induced ER stress and increased GRP78, ATF4, ATF6, p-IRE1, sXBP1, eIF2-α, and CHOP expression levels in gastric tissue. Bax, caspase-3, and caspase-12 levels increased in INDO group, while Bcl-2 level decreased. BCP showed protective activity in gastric tissue and brought these parameters closer to normal levels. In vitro studies revealed that BCP decreased ACh and KCl-induced gastric contractions. Again, BCP decreased gastric acid secretion via M3 receptor pathway but not via H2 and CCK2 receptor pathways. This study revealed that BCP showed healing effects by protecting gastric mucosa from oxidative stress, inflammation, ER stress, and apoptosis in INDO-induced gastric ulcer model. In addition, it was revealed that BCP affects gastric motility by regulating gastric acid secretion via M3 receptor pathway.

## Introduction

Gastric ulcer is a major public health problem today due to its high recurrence rate and the economic losses it causes (Kowada and Asaka 2022). Stomach ulcers are a common health problem worldwide, with an average of 4–6 million people reported to have stomach ulcers each year (Groenen et al. 2009). Ulcer development disrupts the biological balance between defensive and aggressive factors in the gastrointestinal tract, leading to damage to the structure of the gastric mucosa. In this process, increased ambient acidity, decreased mucus secretion, irregular gastric movements, and disruptions in blood flow can trigger erosion of the mucosa and subsequent ulcer formation. Factors that can trigger this condition include non-steroidal anti-inflammatory drugs (NSAIDs), *Helicobacter pylori* (*H. pylori*) infections, and stress factors (Bi 2014; Graham 2014; Kumar et al. 2014). Non-steroidal anti-inflammatory drugs frequently preferred in the treatment of various inflammatory conditions such as rheumatoid arthritis, osteoarthritis, and ischemic cerebrovascular diseases (Goldstein and Cryer 2015; Jeppesen et al. 2020). The therapeutic mechanism of NSAIDs is based on the inhibition of prostaglandin synthesis (Sohail et al. 2023). However, long-term use of these drugs brings serious gastrointestinal (GI) problems. Side effects such as mucosal lesions, gastric ulcers, bleeding, and intestinal inflammation are the main problems frequently encountered in the use of these drugs (Panchal and Prince Sabina 2023). Indomethacin (INDO), an NSAID, exerts its anti-inflammatory effects by inhibiting the cyclooxygenase (COX) enzyme from converting arachidonic acid into prostanoids, the precursors of prostaglandins. However, when used to relieve pain and inflammation, this medicine can cause serious damage to epithelial cells in the digestive tract (Morsy and El-Moselhy 2013; Teichert et al. 2014).

Beta-caryophyllene (BCP) is a natural plant compound belonging to the bicyclic sesquiterpene family. BCP, which is known both as a pure component and as a constituent of plant essential oils, attracts attention with its many beneficial properties. Recent studies have demonstrated the beneficial biological effects of BCP in various organs such as the liver, kidney, and brain. Studies have shown that BCP has antioxidant, anti-inflammatory, antiapoptotic, and anticarcinogenic properties (Gushiken et al. 2022). Thanks to these effects, BCP is known as an important molecule in herbal treatment methods. These receptor types involve multiple signaling pathways in response to mediators such as neurotransmitters, hormones, and other bioactive molecules (Baradaran Rahimi and Askari 2022).

In this study, we aimed to investigate the effects of BCP on INDO-induced gastric ulcer. This study aimed to reveal the effects of BCP application on oxidant damage, endoplasmic reticulum (ER) stress, inflammation, and apoptosis by in vivo method. In addition, it was also aimed to investigate the role of BCP in regulating gastric motility and its effects on gastric acid secretion by in vitro isolated organ bath study method.

## Material and methods

Experimental applications and laboratory analyses in this study were carried out at Atatürk University Medical Experimental Research and Application Center. Ethical approval for the study was obtained from Atatürk University Rectorate Animal Experiments Local Ethics Committee (Decision No: 2022/83).

### Chemicals

Indomethacin (Cat. no. A19910.22), omeprazole (Cat. no. 89352–76-1), beta caryophyllene (Cat. no. 87–44-5), acetylcholine (ACh) (Cat. no. 60–31-1), potassium chloride (KCl) (Cat. no. 7447–40-7), histamine (Cat. no. H7250), famotidine (Cat. no. F6889), atropine (Cat. no. A0132), pentagastrin (Cat. no. C5259); proglumide (Cat. No. M006); chemicals used for Krebs solution: mM: sodium chloride (NaCl) 118, KCl 4.8, magnesium sulfate (MgSO_4_) 1.2, potassium dihydrogen phosphate (KH_2_PO_4_) 1.14, disodium hydrogen phosphate (Na_2_HPO_4_) 15.9, calcium chloride (CaCl_2_) 0.65, glucose 31.6; chemicals used for serosal solution: mM: NaCl 118, KCl 4.8, MgSO_4_ 1.2, KH_2_PO_4_ 1.14, Na_2_HPO_4_ 15.9, CaCl_2_ 0.65, Glucose 31.6; chemicals used for mucosal solution: mM: NaCl 135, KCl 4.8, MgSO_4_ 1.2, CaCl_2_ 1.3, glucose 31.6. In addition, the kits purchased for biochemical analyses; all ELISA kits were purchased from Bostonchem as rat compatible. The antibodies used were purchased from Santa Cruz and Elebscience, and the examinations were carried out.

### Experimental animals and experimental protocols

The experimental animals used in the study were obtained from Atatürk University Medical Experimental Application and Research Center. A total of 116 male adult (2.5–3 months old) Sprague–Dawley rats weighing approximately 240–290 g were used in the study. The daily nutritional and water requirements of the animals in the experimental groups were met ad libitum. The feed content used in rats is humidity 12.8%, crude protein 23%, crude fat 1.7%, crude fiber 3.7%, raw ash 8.3%, sodium 0.5%, manganese 95 mg/kg, iron 31 mg/kg, zinc 95 mg/kg, cobalt 0.5 mg/kg, selenium 0.28 mg/kg, iodine 2.28 mg/kg, vitamin A 12,000,000 IU/kg.

In our study, two separate experimental protocols were applied.

In the first protocol, a total of 96 rats were divided into nine groups as control, indomethacin (INDO) (100 mg/kg), omeprazole (OMP) (5 mg/kg), OMP (5 mg/kg) + indomethacin (INDO) (100 mg/kg), BCP (50 mg/kg) + indomethacin (INDO) (100 mg/kg), BCP (100 mg/kg) + indomethacin (INDO) (100 mg/kg), BCP (200 mg/kg) + indomethacin (INDO) (100 mg/kg), and BCP (200 mg/kg). The control group received only intragastric (i.g.) PBS for 14 days. The INDO group received a single dose of Indomethacin (INDO) (100 mg/kg, i.g.) on the 15 th day of the study (Sengul and Gelen 2019). Omeprazole 5 mg/kg, i.g (El-Ashmawy et al. 2016) and BCP 200 mg/kg, i.g. (Hammad et al. 2018) were administered for 14 days. In the OMP + INDO, BCP50 (He et al. 2021; Francomano et al. 2019; Baradaran and Askari 2022) + INDO, BCP100 (He et al. 2021; Francomano et al. 2019; Baradaran and Askari 2022) + INDO, and BCP200 + INDO groups, a single dose of INDO (100 mg/kg) was administered on day 15, 1 day before the animals were euthanized, following the 14-day treatment period. Animals were sacrificed under general anesthesia 24 h after the last treatment, and gastric tissues were examined macroscopically and placed in appropriate storage conditions for further analyses.

In the second protocol, a total of 20 male rats were used in two groups while performing in vitro studies. In the first group, contraction-relaxation responses of the stomach smooth muscles were determined in 10 rats. In the second group, the effects of BCP on stomach acid secretion activity were investigated in the isolated organ bath environment on 10 rats.

### Macroscopic evaluation of gastric ulcer

Ulcer index and preventive index measurement were calculated using the method used by Küçükler et al. The ulcer score for each group was the average number of ulcers in each group (total number of ulcers/total number of rats). Preventive index = (ulcer index of the ulcer group – ulcer index of the treated group × 100)/ulcer index of the ulcer group. Again, the ulcer scoring in macroscopic findings was evaluated as no (−), mild (+), moderate (+ +) and severe (+ + +) (Küçükler et al. 2022).

### ELISA analyses

Oxidative and antioxidant parameters (MDA, SOD, GPx), proinflammatory (TNF-α, IL-1β) cytokines, iNOS activity, cyclooxygenase-1 (COX-1), and prostaglandin E2 (PGE2) levels in the stomach tissues of rats were measured using the enzyme-linked immunosorbent assay (ELISA) method using BostonChem rat-compatible ELISA kits according to the manufacturer’s protocol (Bolat et al. 2025b).

### Real time PCR analyses

Total RNA was extracted from tissues using RNeasy Mini Kit (Qiagen) according to the manufacturer's instructions. Total mRNA was measured using a NanoDrop spectrophotometer (EPOCH Take3 Plate, Biotek) at 260/280-nm wavelength. Synthesis of cDNA from total RNA was performed using the high capacity cDNA reverse transcription kit (Applied Biosystems, Cat. No. 4368814). Each reaction was performed using 10 μl of RNA. The amount of synthesized cDNA was quantified with a NanoDrop spectrophotometer (EPOCH Take3 Plate, Biotek). The primers required to measure the mRNA transcript levels of the relevant genes by real time PCR were designed by Primer Design program as indicated in Table 1. GRP78, CHOP, ATF6, p-IRE1, sXBP1, Bax, Bcl-2, and caspase 12 mRNA expressions were quantified using SYBR Green Gene Expression Master Mix kit. Amplification and quantification were performed on StepOne Plus Real Time PCR System (Applied Biosystems). Ct values were automatically calculated on the device using the 2 delta threshold cycle (2^−ΔΔCT^) method (Bolat et al. 2025a).

**Table 1:** Gene names and information used in real time PCR analyses

Gen	Primary orientation	Nucleotide sequence	Base length	Catalogue number
GRP78	forward	5'-CATGCAGTTGTGACTGTACCAG-3'	22	NM_013083.2
	reverse	5'- CTCTTATCCAGGCCATATGCAA-3'	22	
ATF6	forward	5’- GTACTGAGGAGACAGCAGCG-3’	20	XM_008769738.4
	reverse	5’- GCCTCTGGTTCTCTGACACC-3’	20	
CHOP	forward	5’- GAAGCCTGGTATGAGGATCT-3’	20	NM_001109986.1
	reverse	5’-GAACTCTGACTGGAATCTGG-3’	20	
XBP-1 s	forward	5’- CTGAGTCCGAATCAGGTGCAG-3’	21	NM_0012271731.1
	reverse	5’- ATCCATGGGGAGATGTTCTGG-3’	21	
p-IRE1	forward	5′-GCAGTTCCAGTACATTGCCATTG-3′	23	NM_001191926.1
	reverse	5′-CAGGTCTCTGTGAACAATGTTGA-3′	23	
BAX	forward	5’- TTTCATCCAGGATCGAGCAG −3’	20	NM_017059.2
	reverse	5’- AATCATCCTCTGCAGCTCCA −3’	20	
β-actin	forward	5’- CAGCCTTCCTTCTTGGGTATG −3’	20	NM_031144.3
	reverse	5’- AGCTCAGTAACAGTCCGCCT −3’	20	
Bcl-2	forward	5’- GACTTTGCAGAGATGTCCAG −3’	20	NM_130422.2
	reverse	5’- TCAGGTACTCAGTCATCCAC −3’	20	
Caspase12	forward	5’- CTGATGATGAAGAGGATGAA −3’	20	NM_130422.2
	reverse	5’- CTGAGGAACTGTAAGCATTA −3’	20	

### Western blot analyses

Protein amounts in stomach tissues were determined according to the protocol of Bolat et al. 200-mg stomach tissue was homogenized in RIPA lysis buffer (sc-24948). Protein measurement was performed with Thermo Pierce™ BCA kit. The prepared gastric tissue samples were loaded onto a gel (Thermo) ready for electrophoresis, respectively. After electrophoresis, proteins were transferred to PVDF membrane. Primary antibodies β-actin (cat. no. E-AB-40338), NF-κB (cat. no. E-AB-32232), caspase 3 (cat. no. sc-7272), ATF4 (cat. no. sc-390063), and eIF2α (cat. no. E-AB-31295) were applied followed by secondary antibody (ab150113). Samples were incubated with the iBind Automated Western System (Thermo). Protein bands were recorded with the iBright™ FL1500 Imaging System (Thermo). Densitometric analysis of the bands was performed with iBright Analysis Software. Each sample was measured at least three times (Bolat et al. 2025b).

### Histopathological and immunofluorescence examination

Stomach tissue samples taken for histological examination were fixed in 10% buffered formaldehyde solution and washed in tap water before tissue tracking was performed. Tracked tissues were blocked in paraffin and 4-µm-thick sections were taken on a microtome device. Then, the sections were stained with hematoxylin–eosin and examined under a light microscope (Olympus BX 51, Japan). Ulcers, edema, hyperemia, degeneration and necrosis seen in the sections were classified as “absent” (−), “mild” (+), “moderate” (+ +), and “severe” (+ + +). In the study, the histopathological images were examined by two pathologists, and the evaluation was done using the blind pathology method (Danisman et al. 2023).

Then the sections were boiled in antigen retrieval (Tris EDTA buffer pH + 6.1, 100 ×) solution. Then protein block was dropped onto the sections. Then primary antibody (Nrf2, Cat No: ab137550) was dropped onto the sections and incubated. Then secondary antibody (FITC, Cat No: ab6785) was dropped. Second primary antibody (HO-1, Cat No: ab13243) was applied, and then immunofluorescence secondary antibody (Texas Red, Cat No: ab6719) was dropped. After the staining process was completed, DAPI (Cat No: D1306) with mounting medium was dropped on the sections. The stained sections were examined using a fluorescence microscope (Zeiss AXIO, GERMANY) (Bolat et al. 2024).

### Effects of BCP on in vitro gastric wall muscle contractions

A contraction-relaxation protocol was established in muscle strips to reveal the role of BCP in the smooth muscles of the stomach wall. The gastric tissues were dissolved in Tyrode’s solution (Krebs solution: mM: sodium chloride (NaCl) 118, potassium chloride (KCl) 4.8, magnesium sulfate (MgSO_4_) 1.2, potassium dihydrogen phosphate (KH_2_PO_4_) 1.14, disodium hydrogen phosphate (Na_2_HPO_4_) 15.9, calcium chloride (CaCl_2_) 0.65, and glucose 31.6, pH: 7.4).

Two or three muscle strips were prepared by taking 1–1.5 cm sections from the stomach tissue. The prepared muscle strips were placed in an organ bath containing 20 ml of Krebs solution containing 95% O_2_, and 5% CO_2_ at approximately 37 °C. The muscle strips placed in the bath were subjected to a 1-g tension and incubation period of 1 h. In our study, two experimental groups, BCP + acetylcholine (ACh) and BCP + KCl, were formed on the gastric muscle wall (Atasever et al. 2016).

#### *BCP* + *ACh group*

10^−7^, 10^−6^, 10^−5^, and 10^−4^ M doses of ACh were applied to the bath, and the response curve was obtained. Control values were determined by averaging the dose–response curve. Then, 250 µg/ml dose of BCP was applied to the bath. After the muscle mass was exposed to BCP for 6 min, 10^−7^, 10^−6^, 10^−5^, and 10^−4^ M doses of ACh were applied to the bath. The responses were recorded and then the tissue was washed three times and rested for 5 min. Using the same protocol, the effect of 500 and 1000 µg/ml doses of BCP was also evaluated, and the effect of different BCP doses on ACh-induced gastric wall smooth muscle motility was determined.

#### *BCP* + *KCI group*

Response curves were generated by applying 20, 40, 60, 80, and 100 mM doses of KCl to the bath. Control values were determined by averaging the response curves. Then, 250-µg/ml dose of BCP was applied. At the end of 6-min exposure to BCP, doses of KCI were applied to the bath. The responses obtained were recorded. The tissue was washed three times and rested for 5 min. BCP doses of 500 and 1000 µg/ml were studied with the same protocol and the effect of increasing doses of BCP on KCI-induced gastric wall smooth muscle contractions was determined.

### Effects of BCP on gastric acid secretion in vitro

Stomach, take serosal solution in (mM: NaCI 118, KCI 4.8, MgSO_4_ 1.2, KH_2_PO_4_ 1.14, Na_2_HPO_4_ 15.9, CaCI_2_ 0.65, glucose 31.6 pH:7.00) and the surrounding connective tissues were cleaned. The stomach was then washed with mucosal solution (mM: NaCI 135, KCl 4.8, MgSO_4_ 1.2, CaCI_2_ 1.3, glucose 31.6 pH:5.00). Then, two plastic cannulas with a diameter of 2 mm were inserted into the pylorus and esophagus. The stomach was then placed in a 20-ml organ bath containing a serosal solution. The temperature of the organ bath was maintained at 37 °C. The bath environment was ventilated with 95% O_2_ and 5% CO_2_ gas. The tissue was incubated for 40–50 min to adapt to the environment and to obtain basal acid secretion. The effects of three different doses of BCP (250 µg/ml, 500 µg/ml, 1000 µg/ml) along with agents that stimulate and inhibit gastric acid secretion by paracrine, hormonal, and neuronal means were evaluated. The effects of these doses on acidic pH were compared with basal pH and changes in ΔpH were calculated. The chemicals used during the experiment were added to the serosal side. Histamine (10^−4^ M/ml) was used to stimulate acid secretion via the paracrine pathway, pentagastrin (10^−^⁶ M/ml) to stimulate it via the hormonal pathway, and acetylcholine (10^−5^ M/ml) to stimulate it via the neuronal pathway. As antagonists of these mechanisms, famotidine (10^−5^ M/ml), proglumide (10^−5^ M/ml), and atropine (10^−^⁶ M/ml) were added respectively. According to the setup shown in Table 2, the pH value of the gastric fluid was measured with different doses of BCP and agonist–antagonist combinations, and the ΔpH value was calculated according to the formula below (Topal and Çelebi 2011).

**Table 2:** BCP experimental groups used in in vivo isolated organ bath

Basal pH group	BCP 250 µg/ml BCP 500 µg/ml BCP 1000 µg/ml	
Histamine group	**A group** (In the presence of proglumide + atropine)	**B group** (In the presence of proglumide + atropine + famotidine)
	A: Histamine B: Histamine + BCP 250 µg/ml C: Histamine + BCP 500 µg/ml D: Histamine + BCP 1000 µg/ml	A: Histamine B: Histamine + BCP 250 µg/ml C: Histamine + BCP 500 µg/ml D: Histamine + BCP 1000 µg/ml
Acetylcholine group	**A group** (In the presence of proglumide + famotidine)	**B group** (In the presence of proglumide + famotidine + atropine)
	A: Acetylcholine B: Acetylcholine + BCP 250 µg/ml C: Acetylcholine + BCP 500 µg/ml D: Acetylcholine + BCP 1000 µg/ml	A: Acetylcholine B: Acetylcholine + BCP 250 µg/ml C: Acetylcholine + BCP 500 µg/ml D: Acetylcholine + BCP 1000 µg/ml
Pentagastrin group	**A group** (In the presence of atropine + famotidine)	**B group** (In the presence of atropine + famotidine + proglumide)
	A: Pentagastrin B: Pentagastrin + BCP 250 µg/ml C: Pentagastrin + BCP 500 µg/ml D: Pentagastrin + BCP 1000 µg/ml	A: Pentagastrin B: Pentagastrin + BCP 250 µg/ml C: Pentagastrin + BCP 500 µg/ml D: Pentagastrin + BCP 1000 µg/ml

### Statistical analysis

The results of biochemical, immunofluorescence, RT-PCR, western blot analyses, and in vitro studies were evaluated using GraphPad Prism 8.0.2 statistics program. For statistical analysis between more than two independent groups, one-way ANOVA followed by Tukey test was applied; *p*<0.05 was considered significant. In histopathological examinations, Shapiro–Wilk test was used to examine whether the histopathological data first showed normal distribution. After, non-parametric Kruskal–Wallis test was used, and Mann–Whitney *U* test was used to determine the differences between the groups.

## Results

### Macroscopic findings

**Fig. 1:**
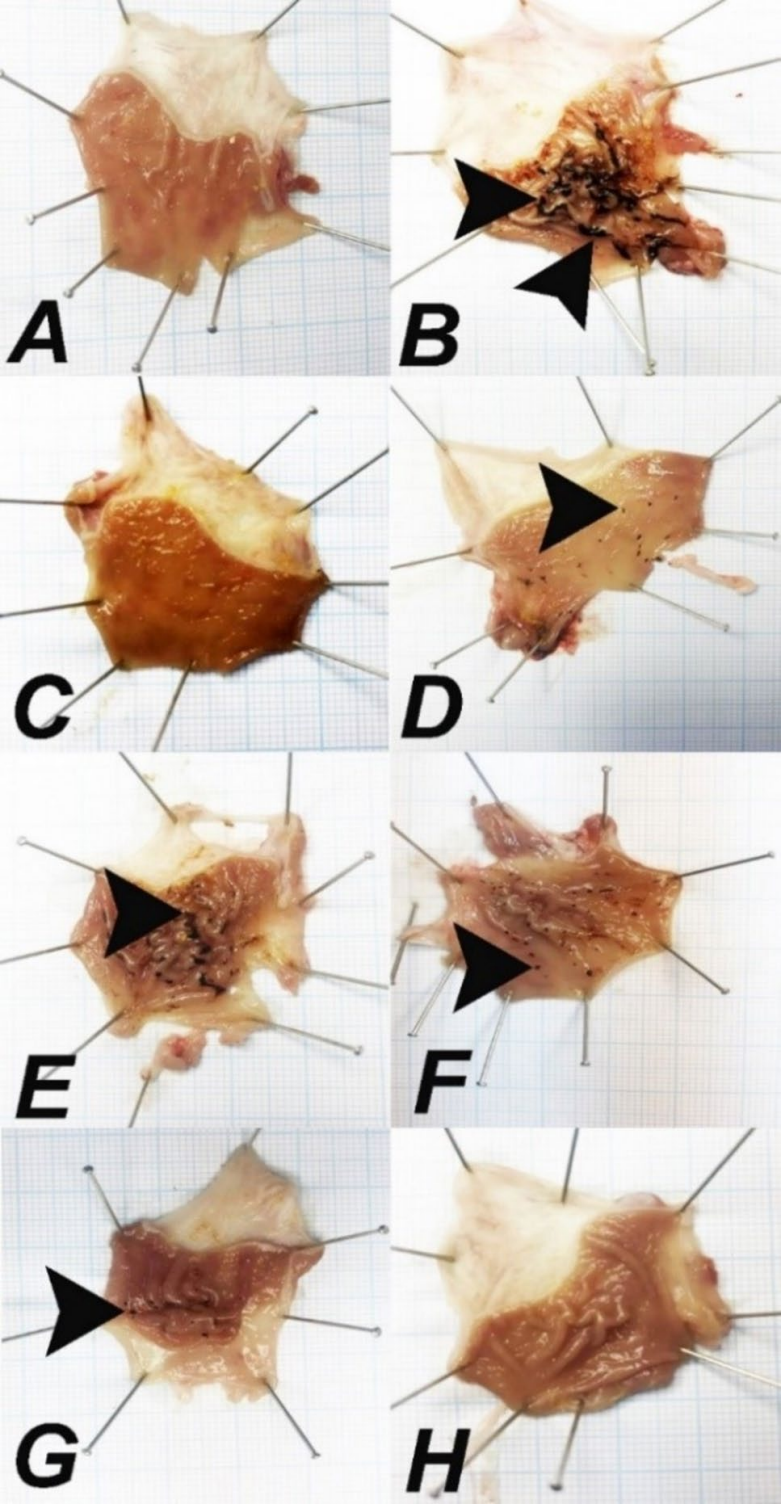
Macroscopic view of the mucosa layer of the gastric tissue of the experimental groups. **A** Control, **B** INDO, **C** OMP, **D** OMP + INDO, **E** BCP50 + INDO, **F** BCP100 + INDO, **G** BCP200 + INDO, **F** BCP200. Bleeding areas in the gastric mucosa are shown with arrowheads

No pathologic findings were observed in the gastric tissues of rats in the control, OMP, and BCP200 groups. In the INDO group, severe erosive-ulcerative foci and bleeding areas were detected in the gastric mucosa. While mild erosion, ulcerative foci and bleeding areas were observed in the gastric mucosa in the OMP + INDO and BCP200 + INDO groups, the analysis revealed a significant difference between these groups and the INDO group (*p* < 0.05). In the BCP50 + INDO group, severe erosive-ulcerative foci and bleeding areas were observed in the gastric mucosa. Moderate erosion, ulceration and bleeding foci were detected in the BCP100 + INDO group, and a significant difference (*p* < 0.05) was found between this group and the INDO group (Fig. 1). The ulcer score, ulcer index and the preventive index of BCP calculated in gastric tissue are presented in Table 3.

**Table 3:** Ulcer score, ulcer index, and preventive index of BCP

	Ulcer score	Ulcer ındex	Preventive ındex
Control	-	-	-
INDO	29.44 ± 2.14^a^	2944	-
OMP	-	-	-
OMP + INDO	2.18 ± 0.57^b^	218	%92.59
BCP50 + INDO	27.51 ± 1.98^a^	2751	%6.55
BCP100 + INDO	13.44 ± 1.13^c^	1344	%54.34
BCP200 + INDO	2.49 ± 0.81^b^	249	%91.54
BCP200	-	-	-

### ELISA analysis findings

**Fig. 2:**
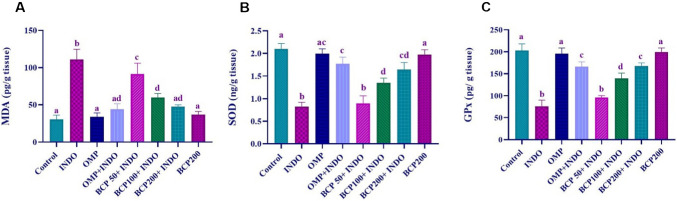
Evaluation of oxidative stress parameters of MDA (**A**), SOD (**B**), and GPx (**C**) in stomach tissue and statistical analysis results. One-way ANOVA and Tukey test (mean ± SD). Different letters (a, b, c, d, ad, and ac) above the columns indicate statistically significant differences between groups (*p* < 0.05)

As a result of the evaluation of oxidative stress parameters in gastric tissue, it was observed that MDA levels significantly increased and SOD and GPx activities decreased in the INDO group compared to the other groups (*p* < 0.0001). Although a decrease in MDA levels and an increase in SOD and GPx activities were found in BCP50 + INDO and BCP100 + INDO groups, these changes were not as effective as in BCP200 + INDO group (*p* < 0.05). The levels of these three parameters were similar in the control, OMP and BCP200 groups and showed favorable results compared to the INDO group (*p* < 0.0001). In the OMP + INDO and BCP200 + INDO groups, there was a decrease in MDA levels and a significant increase in SOD and GPx activities (*p* < 0.05) (Fig. 2).

**Fig. 3:**
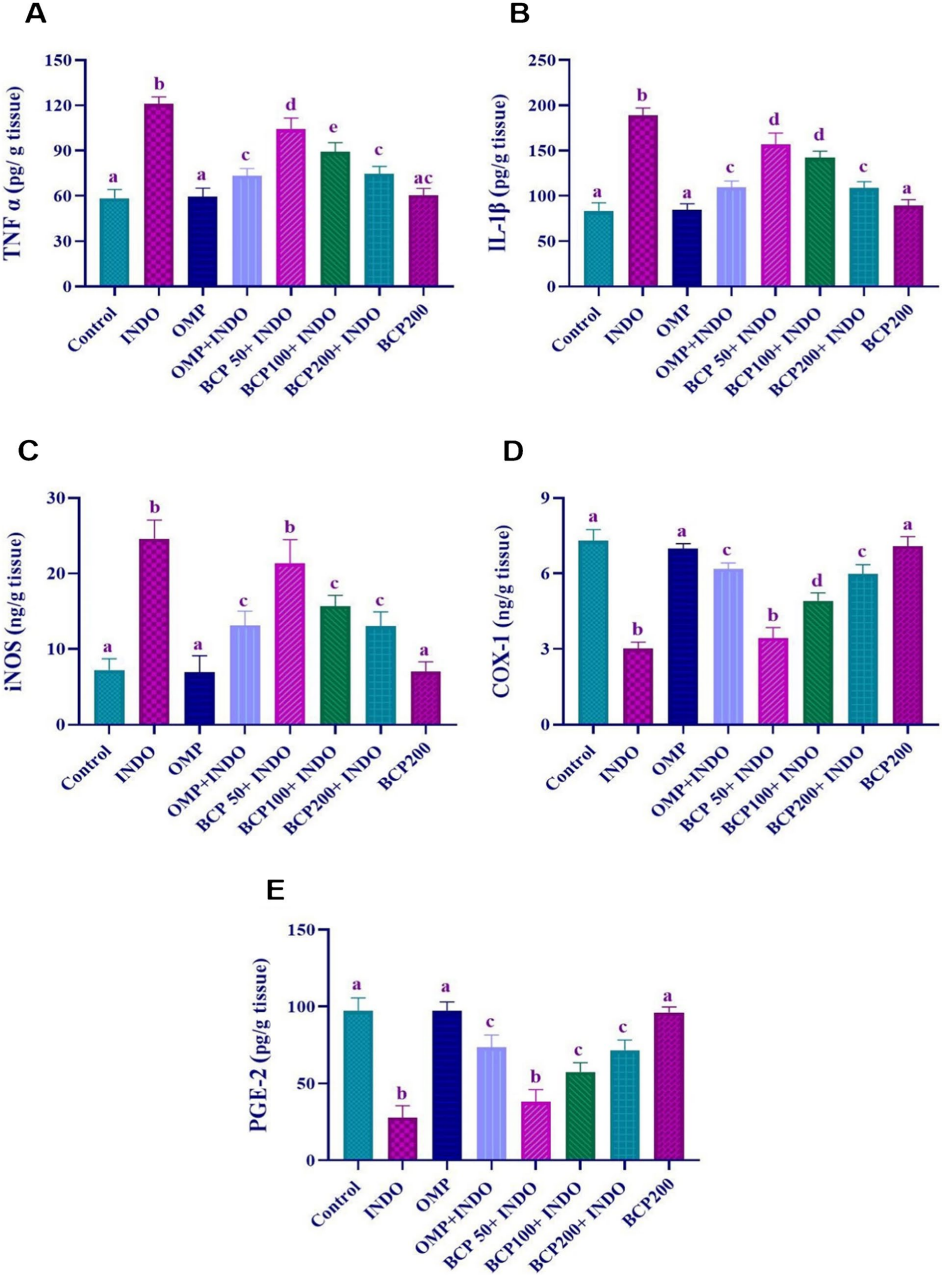
Evaluation of inflammatory parameters of TNF-α (**A**), IL-1β (**B**), iNOS (**C**), COX-1 (**D**) and PGE-2 (**E**) in stomach tissue and statistical analysis results. One-way ANOVA and Tukey test (mean ± SD). Different letters (a, b, c, and d) above the columns indicate statistically significant differences between groups (*p* < 0.05)

As a result of the evaluation of inflammatory mediators in gastric tissues, it was found that TNF-α, IL-1β, and iNOS levels were significantly increased in the INDO group compared to the other groups, while COX-1 and PGE-2 activities were decreased (*p* < 0.0001). TNF-α, IL-1β, iNOS, COX-1, and PGE-2 levels in OMP and BCP200 groups were similar to the control group. In OMP + INDO and BCP200 + INDO groups, TNF-α, IL-1β, and iNOS levels were significantly lower and COX-1, and PGE-2 levels were higher compared to the INDO group (*p* < 0.05). Some decreases were observed in the levels of inflammatory mediators in BCP50 + INDO and BCP100 + INDO groups compared to the INDO group, but these changes were not sufficiently effective compared to the control or other treatment groups (Fig. 3).

### RT-PCR analysis findings

When the expression levels of ER stress-induced chaperone proteins were analyzed, GRP78, CHOP, ATF6, p-IRE1, and sXBP1 expressions were significantly increased in the INDO group compared to the other groups (*p* < 0.0001). There was a decrease in the expression levels of these proteins in BCP50 + INDO and BCP100 + INDO groups compared to the INDO group; however, this decrease was not as effective as the decrease observed in OMP + INDO and BCP200 + INDO groups (*p* < 0.05). In addition, BCP50 + INDO group was not found to be sufficiently effective compared to the control group and other treatment groups. There was no statistically significant difference between the expression levels of OMP, OMP + INDO, BCP200 + INDO, and BCP200 groups (*p* > 0.05).

**Fig. 4:**
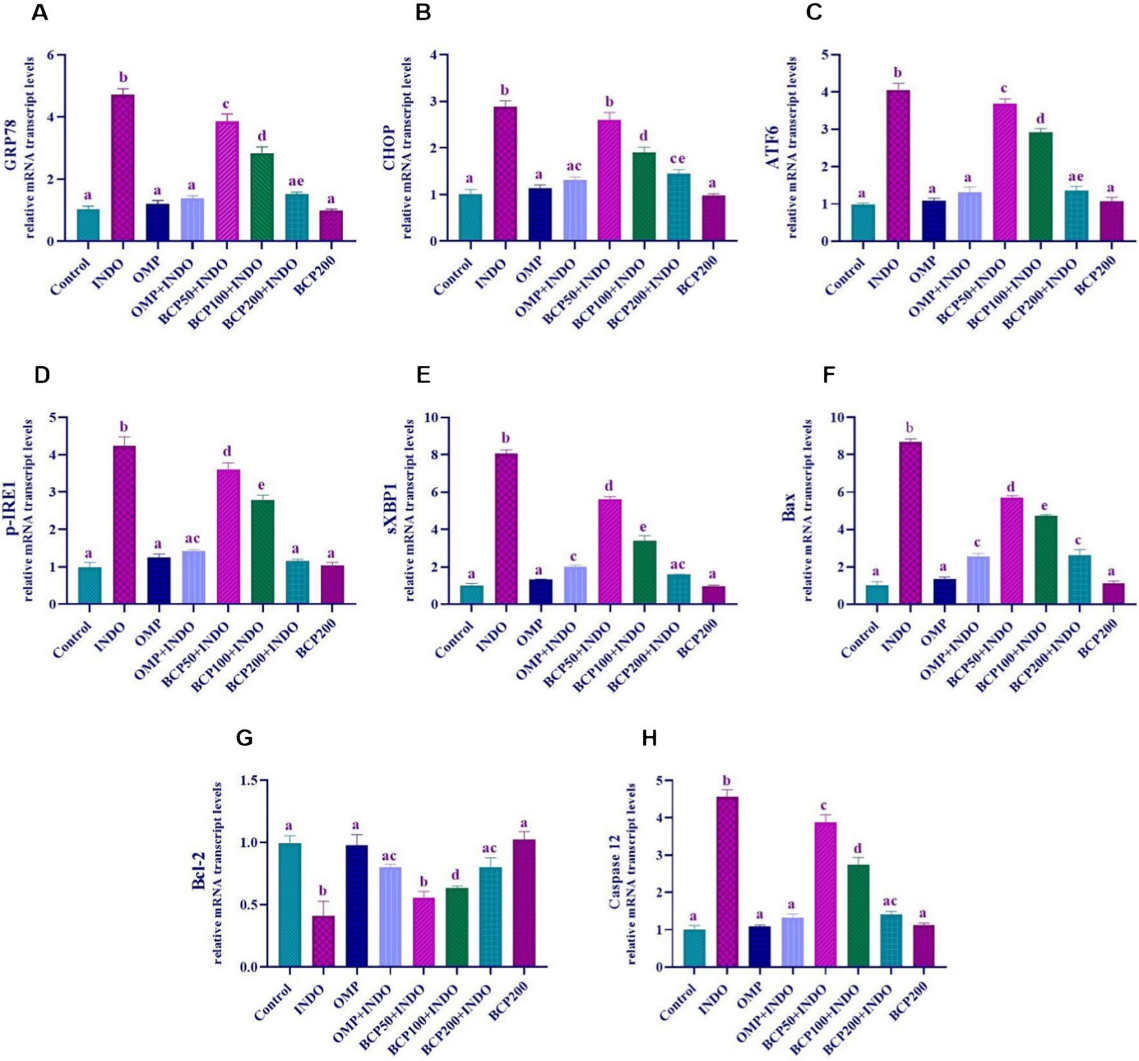
Data and statistical analysis results of ER stress parameters GRP78 (**A**), CHOP (**B**), ATF6 (**C**), p-IRE1 (**D**), and sXBP1 (**E**) and apoptosis parameters Bax (**F**), Bcl-2 (**G**), and caspase 12 (**H**) in stomach tissue. One-way ANOVA and Tukey test (mean ± SD). Different letters (a–d) above the columns indicate statistically significant differences between groups (*p* < 0.05)

When Bax, Bcl-2, and caspase 12 gene expression levels, which are important indicators of apoptosis, were analyzed, it was observed that Bax and caspase 12 expressions increased, while Bcl-2 expression decreased in the INDO group compared to the other groups (*p* < 0.0001). BCP50 + INDO and BCP100 + INDO groups showed a decrease in Bax and caspase 12 expressions compared to the INDO group, but this decrease was not as effective as in OMP + INDO and BCP200 + INDO groups (*p* < 0.05). Bax and caspase 12 levels were also decreased in the BCP50 + INDO group, but this decrease was not found to be effective enough compared to the control group and other treatment groups. Bcl-2 expression increased in BCP50 + INDO and BCP100 + INDO groups compared to the INDO group, but it was not as effective as the increase in OMP + INDO and BCP200 + INDO groups (p < 0.05). No statistically significant difference was observed between Bax, Bcl-2, and caspase 12 expression levels in control, OMP, OMP + INDO, BCP200 + INDO, and BCP200 groups (*p* > 0.05) (Fig. 4).

### Western blot analysis findings

**Fig. 5:**
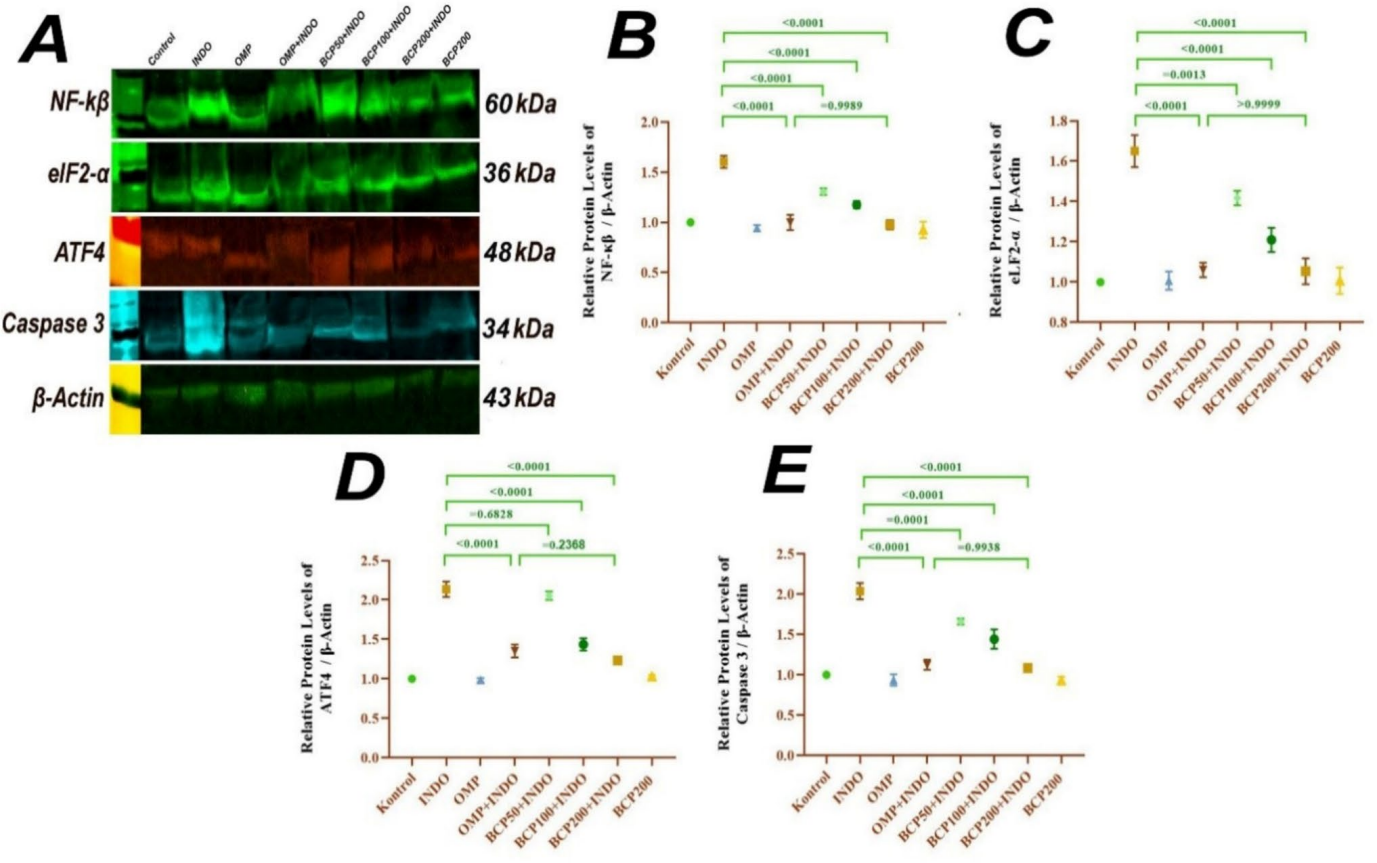
NF-κB, eIF2-α, ATF4, and caspase 3 protein levels in stomach tissue Western blot analysis results. Protein bands obtained as a result of blotting (**A**), NF-κB analysis results (**B**), eIF2-α analysis results (**C**), ATF4 analysis results (**D**), and caspase 3 analysis results (**E**). One-way ANOVA and Tukey test (mean ± SD)

NF-κB, elF2-α, ATF4, and caspase 3 protein levels were analyzed by Western blot analysis. As shown in Fig. 5, these protein levels were significantly increased in the INDO group compared to the other groups (*p* < 0.0001). BCP50 + INDO group showed a decrease in NF-κB, caspase 3 (*p* < 0.0001), elF2-α (*p* = 0.0013), and ATF4 (*p* = 0.6828) expression levels compared to the INDO group. There was a significant decrease in NF-κB, elF2-α, ATF4, and caspase 3 protein levels in OMP200 + INDO, BCP100 + INDO, and BCP200 + INDO groups compared to the INDO group (*p* < 0.0001) (Fig. 5).

### Histopathological and immunofluorescence findings

**Fig. 6:**
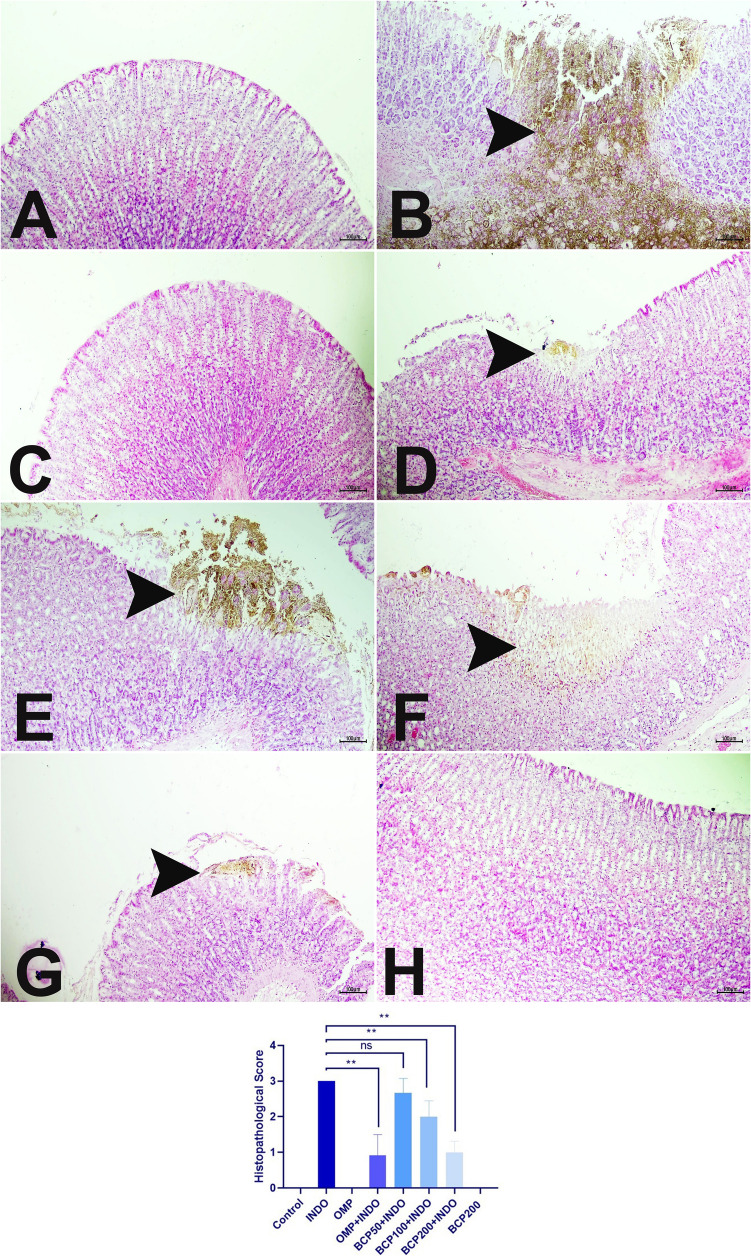
Gastric mucosal tissue, **A** control, **B** INDO, **C** OMP, **D** OMP + INDO, **E** BCP50 + INDO, **F** BCP100 + INDO, **G** BCP200 + INDO, **H** BCP200. Necrotic areas in the gastric mucosa are shown with arrowheads. H&E, Bar: 100 µm. Scoring of histopathological findings and statistical analysis data. (**: *p* < 0.05, ns: no standard deviation). Kruskal–Wallis and Mann–Whitney *U* test (mean ± SD)

In the control, OMP, and BCP200 groups, normal histologic structure was observed when the gastric tissues were examined histopathologically. In the INDO group, severe necrotic mass was showed. There was severe erosion and ulceration in the mucosal layer. Although these findings decreased in the BCP50 + INDO group, this was not statistically significant (*p* > 0.05). In the OMP + INDO, BCP100 + INDO, and BCP200 + INDO groups, these findings were significantly reduced compared to the INDO group (*p* < 0.05) (Fig. 6).

**Fig. 7:**
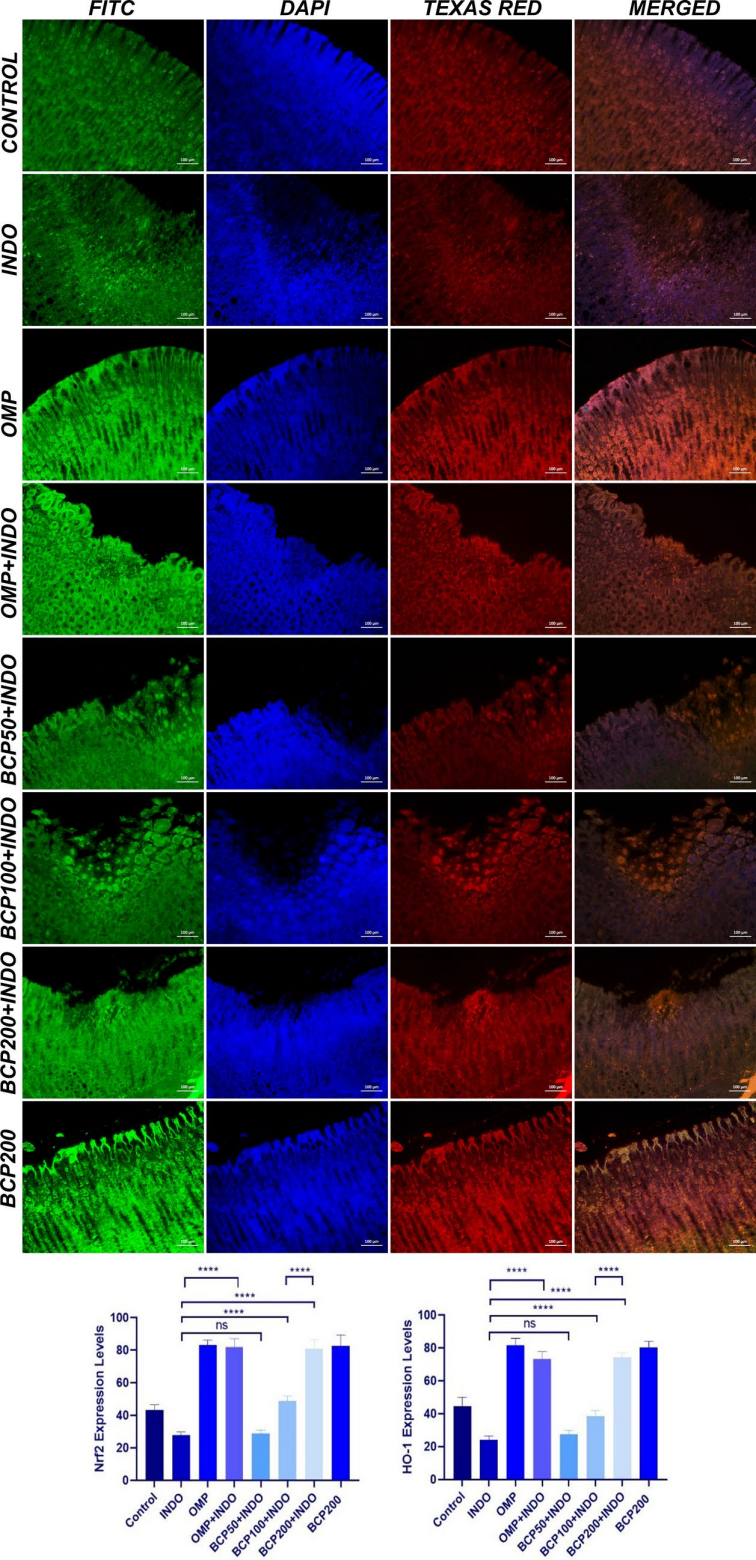
Gastric tissue, Nrf2 (FITC) and HO-1 expressions in mucosal epithelial cells (Texas Red), DAPI; nuclear stain, Merged; FITC, DAPI and Texas Red combination. IF, Bar: 100 µm. (****: *p* < 0.0001, ns: no standard deviation). One-way ANOVA and Tukey test (mean ± SD)

According to the immunofluorescence staining results, moderate levels of Nrf2 and HO-1 expressions were detected in the gastric tissue in the control group. In the INDO group, mild levels of Nrf2 and HO-1 expressions were observed in the cells around the necrotic areas in the mucosa layer. Severe levels of Nrf2 and HO-1 expressions were found in the OMP, OMP + INDO, BCP200 + INDO, and BCP200 groups, and a statistically significant difference was found when compared to the INDO group (*p* < 0.0001). Mild levels of Nrf2 and HO-1 expressions were detected in the BCP50 + INDO group, and no significant difference was observed when compared to the INDO group (*p* > 0.05) (Fig. 7).

### Findings on the contractile and relaxant effects of BCP on the stomach wall smooth muscles

#### *In the BCP* + *ACh (n* = *5) group*

**Fig. 8:**
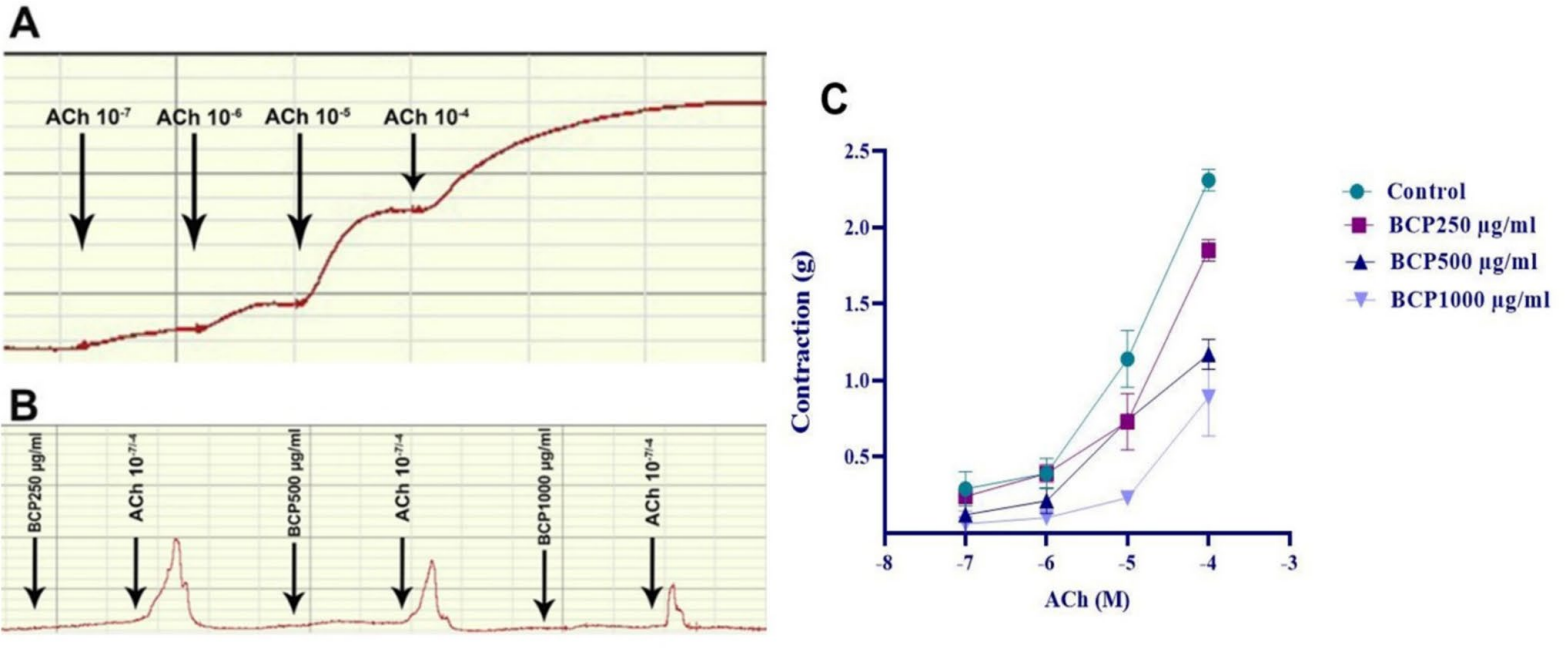
Results of statistical analysis of data obtained from the contraction response of muscle strips to ACh after BCP was applied to the in vitro organ bath at the determined doses. Only ACh application (**A**), treatment with different doses of BCP (**B**) and statistical analysis (**C**). One-way ANOVA and Tukey test (mean ± SD)

10^−^⁷, 10^−^⁶, 10^−5^, and 10^−4^ M doses of ACh were cumulatively added to the bath, and the dose–response curve of ACh was analyzed (Fig. 8A). Then, after administration of ACh doses, the effects of 250-, 500-, and 1000-µg/ml BCP doses on contraction and relaxation were recorded (Fig. 8B). In the presence of a 250-µg/ml dose of BCP, no significant change in the ACh-induced contractile response was observed, whereas 500- and 1000-µg/ml BCP doses significantly reduced the contractile response to ACh (Fig. 8).

#### *In the BCP* + *KCI (n* = *5) group*

**Fig. 9:**
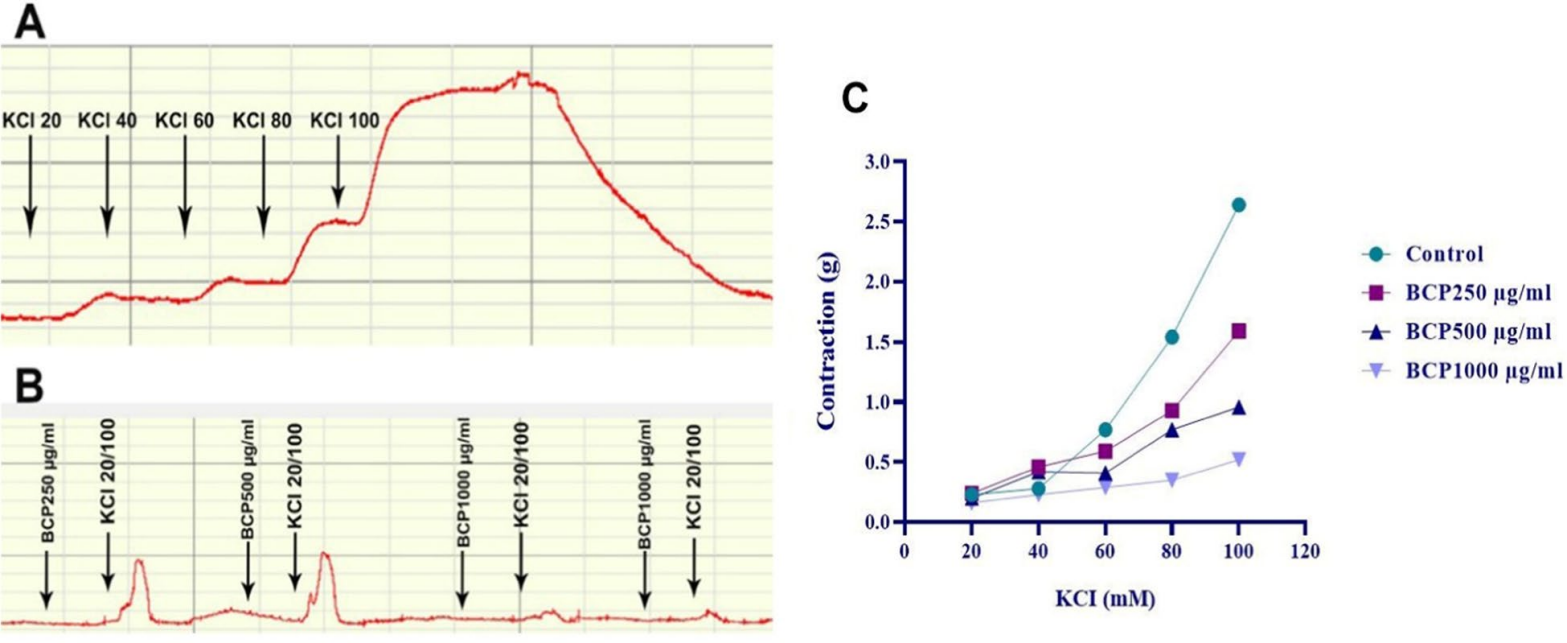
Results of statistical analysis of data obtained from the contraction response of muscle strips to KCI after BCP was applied to the in vitro organ bath at the determined doses. Only KCI application (**A**), treatment with different doses of BCP (**B**) and statistical analysis (**C**). One-way ANOVA and Tukey test (mean ± SD)

KCl concentrations of 20, 40, 60, 80, and 100 mM were added cumulatively to the bath medium, and the dose–response curve of these concentrations was analyzed (Fig. 9A). Then, changes in contraction and relaxation caused by 250-, 500-, and 1000-µg/ml BCP doses were recorded in response to KCl doses (Fig. 9B). While no significant change was observed in the contraction response caused by KCl in the presence of 250-µg/ml BCP dose, 500 and 1000 µg/ml BCP doses significantly reduced the contraction response to KCl (Fig. 9).

### Effect of BCP on histamine-ınduced gastric acid secretion in ısolated rat stomach tissue

**Fig. 10:**
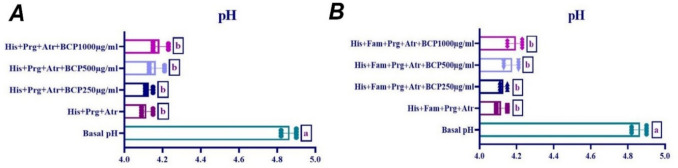
Effect of BCP on histamine-induced gastric acid secretion (**A**: in the presence of proglumide (Prg) and atropine (Atr), **B**: in the presence of proglumide, famotidine (Fam) and atropine (one-way ANOVA and Tukey test, mean ± SD). Different letters (a, b) above the columns indicate statistically significant differences between groups (*p* < 0.05)

The effects of 250-µg/ml, 500-µg/ml, and 1000-µg/ml doses of BCP on histamine-induced gastric acid secretion were investigated. When 10^−4^ M dose of histamine was administered with proglumide and atropine, a significant increase in gastric acid secretion was observed. As shown in Fig. 10, no statistically significant difference was observed in His + BCP 250 µg/ml, His + BCP 500 µg/ml, and His + BCP 1000 µg/ml groups in histamine-induced gastric acid secretion (*p* > 0.05). Furthermore, when the effect of BCP on histamine-induced gastric acid secretion in the presence of proglumide, atropine, and famotidine was evaluated, no difference was observed between the groups (*p* > 0.05) (Fig. 10).

### Effect of BCP on acetylcholine-ınduced gastric acid secretion in ısolated rat stomach tissue

**Fig. 11:**
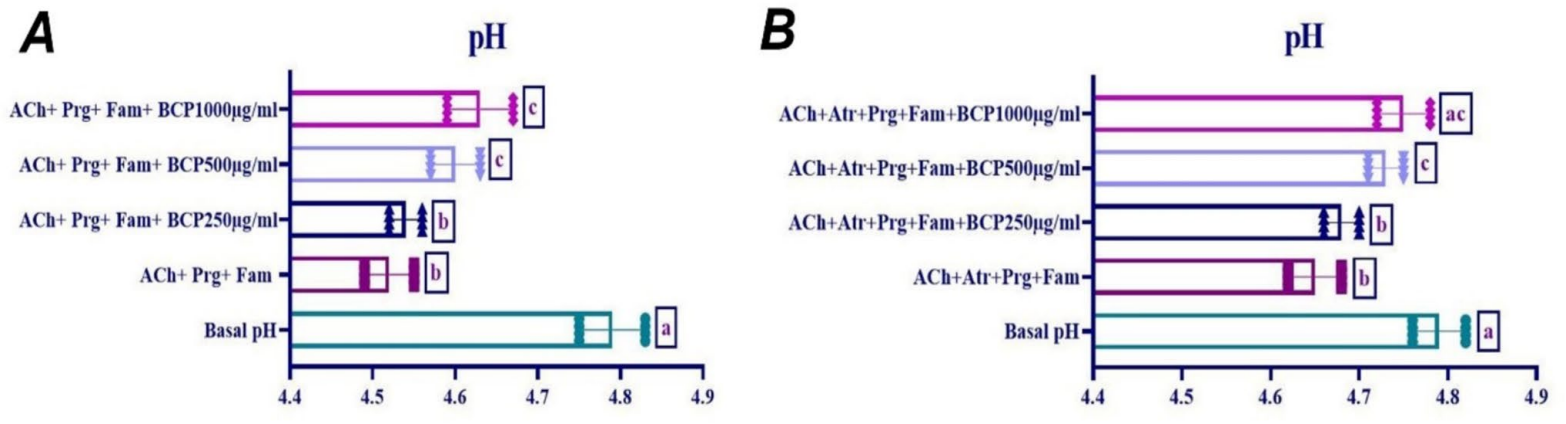
Effect of BCP on ACh-induced gastric acid secretion (**A**: in the presence of proglumide (Prg) and famotidine (Fam), **B**: in the presence of proglumide, famotidine, and atropine (Atr). (One-way ANOVA and Tukey test, mean ± SD). Different letters (a–c) above the columns indicate statistically significant differences between groups (*p* < 0.05)

The effects of 250-µg/ml, 500-µg/ml, and 1000-µg/ml doses of BCP on ACh-induced gastric acid secretion were examined. It was observed that 10^−4^ M dose of ACh caused an increase in gastric acid secretion in the presence of proglumide and atropine. As shown in Fig. 11, while there was no difference in ACh + BCP 250 µg/ml group (*p* > 0.05), gastric acid secretion was significantly decreased in ACh + BCP 500 µg/ml and ACh + BCP 1000 µg/ml groups (*p* < 0.05). Furthermore, when the effect of BCP on ACh-induced gastric acid secretion in the presence of proglumide, famotidine, and atropine was evaluated, it was found that gastric acid secretion was significantly decreased in ACh + BCP 500 µg/ml and ACh + BCP 1000 µg/ml groups (*p* < 0.05) (Fig. 11).

### Effect of BCP on pentagastrin-ınduced gastric acid secretion in ısolated rat stomach tissue

**Fig. 12:**
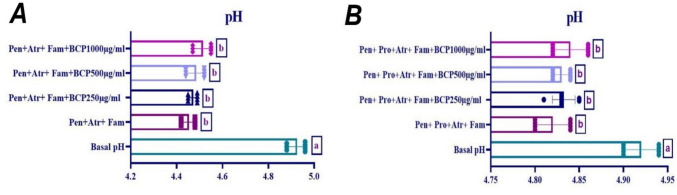
Effect of BCP on pentagastrin-induced gastric acid secretion. **A**: in the presence of atropine (Atr) and famotidine (Fam), **B**: in the presence of proglumide (Prg), famotidine, and atropine (one-way ANOVA and Tukey test, mean ± SD). Different letters (a, b) above the columns indicate statistically significant differences between groups (*p* < 0.05)

The effect of 250-µg/ml, 500-µg/ml, and 1000-µg/ml doses of BCP on pentagastrin-induced gastric acid secretion was investigated. It was observed that 10^−4^ M dose of pentagastrin increased gastric acid secretion in the presence of proglumide and atropine. As shown in Fig. 12, no dose of BCP altered pentagastrin-induced acid secretion, and no statistically significant difference was found (*p* > 0.05). Furthermore, when the effect of BCP on pentagastrin-induced gastric acid secretion in the presence of proglumide, famotidine, and atropine was evaluated, no significant difference was observed between the groups (*p* > 0.05) (Fig. 12).

## Discussion

Ulcers are open wounds or lesions characterized by tissue loss, which can occur on any mucosal surface. Gastric ulcers, in particular, may progress to deeper layers under the influence of digestive fluids such as gastric acid and pepsin (Beiranvand 2022). They typically form when the gastric mucosa’s defense mechanisms are insufficient against aggressive factors like acid, pepsin, NSAID overuse, *H. pylori* infections, stress, alcohol, and smoking (Zaghlool et al. 2015; Al-Sayed and El-Naga 2015). Among NSAIDs, INDO has a high potential to cause gastric ulcers by inhibiting enzymes that reduce prostaglandin synthesis, leading to both its anti-inflammatory and harmful effects (Hilário et al. 2006).

Reactive oxygen species (ROS) are natural by-products of metabolism, and their excess causes oxidative stress. Antioxidant enzymes like superoxide dismutase (SOD), glutathione peroxidase (GPx), and catalase neutralize ROS to maintain cellular balance. However, when ROS production surpasses antioxidant capacity, cellular damage occurs (Sies et al. 2022). Lipid peroxidation damages cell membranes, and NSAIDs increase oxidative stress by promoting lipid peroxidation, protein oxidation, and hydroxyl radical production (Naito and Yoshikawa 2006). Studies show that INDO raises MDA levels in gastric tissue and induces ulcers (Küçükler et al. 2022; LIU et al. 2015). INDO also decreases SOD activity (Pineda-Peña et al. 2018; Boyacioglu et al. 2016) and inhibits GPx activity, worsening oxidative stress (Ighodaro and Akinloye 2018). BCP, a natural antioxidant, suppresses oxidative stress and restores SOD and GPx activities (Gushiken et al. 2022). This study found that BCP alleviated oxidative stress caused by INDO, normalizing MDA, SOD, and GPx levels.

The NRF2/HO-1 signaling pathway protects against oxidative stress by increasing antioxidant enzymes like SOD and GPx (Yanaka 2018). NRF2, a key transcription factor, is normally bound to KEAP1 in the cytoplasm and degraded via the proteasome (El Asar et al. 2019). During oxidative stress, ROS modify KEAP1, releasing NRF2, which then translocates to the nucleus, binds to AREs, and promotes antioxidant enzyme expression, including HO-1 (Seok et al. 2022). These enzymes neutralize ROS and reduce MDA, thus enhancing antioxidant defenses and protecting cells (Chowdhury et al. 2022). In INDO-induced gastric ulcer studies, decreased expression of NRF2 and HO-1 has been shown to contribute to increased oxidative stress and mucosal damage (Jia et al. 2023). BCP has been shown to counteract this by activating NRF2/HO-1 signaling and inhibiting ferroptosis (Hu et al. 2022). In this study, INDO suppressed NRF2/HO-1 activity, but BCP treatment restored this activity in a dose-dependent manner, reversing the inhibition seen in the INDO group.

Inflammation is a natural immune response, and cytokines play a crucial role in initiating and regulating this process (Opal and DePalo 2000). Inflammatory cytokines such as TNF-α, IL-1β, and IL-6 have important roles in the inflammation process (Ng et al. 2018). INDO promotes gastric ulcers by increasing ROS and proinflammatory cytokines in gastric tissue (Shim and Kim 2016; Hermenean et al. 2017). In addition, it has been reported that NF-κB levels increase with the increase in TNF-α (Khodir et al. 2024). Conversely, BCP has shown anti-inflammatory effects, suppressing cytokines like NF-κB, TNF-α, and IL-1β (Baradaran Rahimi and Askari 2022; Yeom et al. 2022). In this study, BCP dose-dependently reduced these cytokine levels compared to the INDO group, indicating its protective role in the gastric mucosa via inflammation suppression.

Nitric oxide (NO) plays a vital role in protecting and repairing the gastric mucosa by regulating blood flow, balancing acid–base secretion, and supporting mucus production (Ismail Suhaimy et al. 2017). In a gastric mucosal injury model induced by INDO, it has been reported that iNOS levels significantly increase in INDO groups (Zheng et al. 2016). Prostaglandin E2 (PGE2) is another key mediator in maintaining gastric mucosal integrity. Its synthesis begins with the conversion of arachidonic acid to PGH2 via COX-1 and COX-2, and then to PGE2 through prostaglandin E synthases (Kloska et al. 2020). A decrease in PGE2 is associated with mucosal injury and ulcer progression (Adhikary et al. 2012). COX-1 is constitutively expressed and supports normal gastric function, whereas COX-2 is inducible during inflammation (Laine et al. 2008). INDO, a nonselective COX inhibitor, suppresses both COX-1 and COX-2, leading to reduced PGE2 levels and compromised mucosal defense (Zhang et al. 2021). In this study, INDO significantly reduced PGE2 and COX-1 activity, while increasing NOS. However, BCP treatment reversed these effects in a dose-dependent manner, increasing PGE2 and COX-1 and decreasing NOS. These findings suggest that BCP may protect the gastric mucosa by modulating the PGE2/COX-1/NOS pathway, mitigating inflammation and ulcer formation.

The endoplasmic reticulum performs N-linked glycosylation, an example of post-translational modifications, and functions as an important hub in cellular signaling processes. However, disruption of these functions of the ER under pathological conditions can lead to a condition defined as “ER stress” (Chaudhari et al. 2014). To counteract this, cells activate the unfolded protein response (UPR), primarily regulated by three transmembrane proteins: PERK, ATF6, and IRE1. These proteins are normally inactivated by GRP78, which dissociates under stress to initiate UPR signaling (Bertolotti et al. 2000). Studies show that INDO increases GRP78 expression, indicating ER stress in gastric tissues (Boonyong et al. 2020). Activation of PERK leads to eIF2α phosphorylation, which suppresses general protein synthesis but promotes translation of stress-related genes such as ATF4. ATF4 regulates genes involved in redox balance, amino acid metabolism, and stress adaptation. However, it also induces CHOP, a pro-apoptotic factor that mediates ER stress-induced apoptosis during prolonged stress (Vattem and Wek 2004; Brewer 2014; Alshammari et al. 2021). Wongsakul et al. (2022) confirmed that INDO increases eIF2α, ATF4, and CHOP expression. In contrast, BCP has been shown to suppress GRP78, ATF4, and CHOP, alleviating ER stress (Wongsakul et al. 2022).

Similarly, ATF6, upon dissociation from GRP78, translocates to the nucleus and upregulates ER stress–related genes such as CHOP, GRP78, GRP94, p-IRE1, and XBP1, enhancing the UPR and promoting cell survival (Ibrahim et al. 2019; Chuan et al. 2021). INDO was shown to upregulate ATF6, p-IRE1, and XBP1 (Ainiwaer et al. 2023), while BCP downregulated ATF6 expression (Proshkina et al. 2020). Consistent with these findings, this study showed that INDO significantly increased GRP78, ATF4, CHOP, eIF2α, ATF6, p-IRE1, and XBP1 expression, while BCP treatment reversed these effects in a dose-dependent manner, suggesting its protective role against ER stress-mediated damage.

Apoptosis involves a decrease in anti-apoptotic Bcl-2 and an increase in pro-apoptotic Bax, leading to enhanced mitochondrial permeability and cytochrome c release, which subsequently activates caspase-3. This enzyme drives the degradation of cellular components in the final stages of cell death. Under ER stress, apoptosis becomes more complex with the activation of caspase-12, triggered by misfolded protein accumulation in the ER. Caspase-12 promotes ER-specific apoptosis and supports caspase-3 activation via mitochondrial pathways (Ouyang et al. 2021; Tosun et al. 2021). Studies report that INDO-induced ER stress in gastric tissue decreases Bcl-2 while increasing Bax and caspase-3 levels, promoting apoptosis (Bank et al. 2008). Conversely, BCP has been shown to inhibit apoptosis by increasing Bcl-2 and reducing Bax expression (Zhang et al. 2017). In this study, INDO-induced gastric ulceration resulted in excessive caspase-3 activation, elevated Bax, reduced Bcl-2, and increased caspase-12, indicating ER stress–mediated apoptosis. BCP treatment reversed these effects in a dose-dependent manner, highlighting its protective role against ER stress–induced apoptosis.

Most of the effects of BCP in gastric tissue are related to its antioxidant, anti-inflammatory and antiapoptotic activities (Tambe et al. 1996). This study investigated the impact of BCP on KCl-induced smooth muscle contractions of rat gastric wall in vitro. At 250 µg/ml, BCP had no significant effect on contractions, while 500 µg/ml and 1000 µg/ml doses significantly suppressed muscle contractions in a dose-dependent manner. These inhibitory effects are likely mediated via voltage-sensitive L-type Ca^2^⁺ channels, as KCl-induced depolarization increases Ca^2^⁺ influx through these channels, leading to contraction (Petkova et al. 2000). BCP’s suppression of this mechanism suggests it may inhibit K⁺-induced depolarization and calcium entry, thereby reducing smooth muscle contractility.

In this study, cumulative doses of acetylcholine (ACh) (10⁻⁷ to 10⁻^4^ M) were applied to gastric tissue to generate a dose–response curve. While 250-µg/ml BCP had no significant effect on ACh-induced smooth muscle contractions, 500-µg/ml and 1000-µg/ml doses significantly suppressed contractions in a dose-dependent manner. The ACh-induced contraction of gastric smooth muscle is primarily mediated via M3 receptors, activating both phospholipase C (PLC)/IP3 signaling and voltage-sensitive L-type Ca^2^⁺ channels (Ishii and Kurachi 2006). Binding of ACh to M3 receptors triggers IP3-mediated Ca^2^⁺ release from the ER and promotes Ca^2^⁺ influx through L-type channels, leading to muscle contraction (Stengel et al. 2002). The inhibitory effects observed suggest that BCP at higher concentrations attenuates smooth muscle contractions by blocking both IP3-mediated intracellular Ca^2^⁺ release and L-type Ca^2^⁺ channel-dependent influx, thereby reducing intracellular calcium levels.

HCl secretion in the stomach occurs via three pathways. These pathways are paracrine stimulation with histamine, neuronal stimulation with acetylcholine, and hormonal stimulation with gastrin (Schubert and Peura 2008; Bitziou et al. 2008). In this study, histamine (H2 receptor agonist), acetylcholine (M3 receptor agonist), and pentagastrin (CCK2 receptor agonist) pathways were examined in isolated rat stomach.

Histamine, released by ECL cells, binds to H2 receptors on parietal cells, activating adenylate cyclase, increasing cAMP, and stimulating H⁺,K⁺-ATPase, resulting in HCl secretion (Chew et al. 1980). In this study, histamine significantly decreased gastric pH, indicating increased acid secretion. However, BCP had no inhibitory effect on histamine-induced acid secretion, suggesting it does not interfere with this paracrine pathway.

Acetylcholine stimulates acid secretion by binding to M3 receptors on both parietal and ECL cells, leading to IP3-Ca^2^⁺ signaling, PLC activation, and ultimately H⁺,K⁺-ATPase stimulation (Stengel et al. 2002; Ishii and Kurachi 2006). The study found that BCP significantly reduced ACh-induced acid secretion, implying a possible interaction with M3 receptors, potentially affecting both neurogenic and paracrine pathways. This suggests BCP may regulate gastric acid production by modulating acetylcholine-mediated signaling.

Pentagastrin, a CCK2 receptor agonist, stimulates acid secretion by acting on parietal and ECL cells (Schubert and Peura 2008). However, BCP had no significant effect on pentagastrin-induced secretion, suggesting it does not affect CCK2 receptors.

These findings are supported by Ybañez-Julca et al. (2023), who reported that *Valeriana pilosa* essential oil, which contains β-caryophyllene (BCP), exerted antispasmodic effects by inhibiting muscarinic receptors and calcium channels. It was suggested that BCP binds strongly to M2/M3 muscarinic receptors and voltage-gated calcium channels, aligning with our results showing selective inhibition of ACh-stimulated acid secretion.

## Conclusion

In this study, the protective efficacy of BCP against INDO-induced gastric ulcer in rats was demonstrated by in vitro and in vivo methods. In this study, the protective effects of BCP against oxidative stress, ER stress, inflammation, and apoptosis in the stomach tissue induced by INDO were determined in vivo. Again, as a result of in vitro studies, BCP has been proven to show protective effects both by suppressing gastric acid secretion and muscle contraction and by cellular mechanisms in gastric ulcer model. Therefore, it is predicted that BCP can be used as a powerful gastric protector in daily life.

## Data Availability

All source data for this work (or generated in this study) are available upon reasonable request.
